# Treatment options for isolated aortic valve insufficiency: a review

**DOI:** 10.3389/fcvm.2024.1381102

**Published:** 2024-04-11

**Authors:** Salem Argaw, Nasim Azizgolshani, David Blitzer, Hiroo Takayama, Isaac George, Luigi Pirelli

**Affiliations:** Division of Cardiovascular Surgery, Department of Surgery, Columbia University Irving Medical Center, New York, NY, United States

**Keywords:** aortic insufficiency, aortic regurgitation, aortic valve replacement, aortic valve repair, transcatheter management of AI, aortic prosthesis

## Abstract

Aortic insufficiency (AI) is a valvular disease with increasing prevalence in older patients. The modern era provides numerous options for the management of AI which is explored here. Traditional interventions included aortic valve replacement with either mechanical or bioprosthetic aortic valves. While the former is known for its durability, it has grown out of favor due to the potential complications of anticoagulation. The preference for bioprosthetic valves is thus on the rise, especially with the advancements of transcatheter technology and the use of valve-in-valve therapy. Surgical options are also no longer limited to replacement but include complex techniques such as those required for aortic valve repair, Ozaki neocuspidization, Ross procedure and valve-sparring aortic root repair. Transcatheter options for the management of AI are not commercially available currently; however, preliminary data from ad-hoc trials, showed promising results and potential use of transcatheter technology in a variety of patients with pure AI.

## 1 Introduction

The prevalence of moderate or greater, aortic insufficiency (AI) increases with age. The Framingham Heart Study found a prevalence rate of 0.5% in men and 0.2% in women of age 50–59 years compared to 2.2% in men and 2.3% in women of age 70–83 years (1). Other studies have quoted a prevalence rate of moderate or severe AI of 1.6%, and even as high as 4.5%, in patients over 65 years old (2, 3). Therefore, in an increasingly aging population, the management of AI is an important discussion.

The etiologies of AI can be grouped under the umbrella categories of leaflet disease or aortopathy. The former is most commonly seen with degenerative, congenital, infectious, and traumatic causes of structural damage to the aortic valve; while acute dissections or aneurysms—as frequently seen in patients with connective tissue or autoimmune disorders—lead to disruption or dilatation of the aortic root (4). In low- to middle-income countries, rheumatic heart disease is the leading cause of AI, while root dilation is the culprit in many high-income countries (5).

Presentation and natural history of AI can vary greatly depending on acuity. Acute AI, most frequently seen with aortic dissections, endocarditis, or trauma, leads to sudden increase in left ventricular volume and end-diastolic pressure leading to pulmonary edema (6). These patients present in cardiogenic shock or heart failure as forward stroke volume decreases acutely and therefore, require urgent intervention. Patients with chronic AI, however, can remain asymptomatic for many years as the heart compensates by remodeling. When the heart can no longer accommodate the increased pressure and volume in the left ventricle, patients develop progressive dyspnea on exertion and reduced exercise tolerance in addition to widened pulse pressure on exam and increased left ventricle end-systolic dimension (LVESD) on imaging. LVESD >50 mm has been associated with a 19% likelihood of death, symptoms, or LV dysfunction (7).

The 2022 ACC/AHA Guideline for the Diagnosis and Management of Patients with Valvular Heart Disease provides a Class I indication for surgical intervention in patients with symptomatic severe AI, asymptomatic severe AI with ejection fraction of less than or equal to 55%, as well as in patients with severe AI who are already undergoing cardiac surgery (8). Additionally, there is a Class IIa indication for intervention in patients who are asymptomatic with severe AI and normal ejection fraction but with LVESD >50 mm or indexed LVESD >25 mm/m^2^ (8). Current management technique includes a variety of surgical replacement and repair options. While commercially available transcatheter valves are not currently approved for the treatment of AI, novel technologies and devices are being developed with the goal of representing a valid option to surgery in high-risk patients.

## 2 Surgical management of aortic insufficiency

### 2.1 Without presence of aortic root dilation

#### 2.1.1 Aortic valve replacement

Surgical aortic valve replacement (SAVR) has been the mainstay of treatment for patients with AI. The choice of prosthesis is an individualized shared decision that should consider the patient's age, lifestyle, pattern of adherence, preference, life expectancy, and the valve's durability. SAVR outcomes for AI have shown encouraging results with a recent national study of >12,000 patients showing an operative mortality of 1.1% (9).

##### 2.1.1.1 Mechanical and bioprosthetic valve replacement

Mechanical prosthetic valves are well accepted for their durability; however, there has been a progressive shift towards bioprosthetic valves among patients to avoid the hassle and complication of anticoagulants. Studies quote a rise in bioprosthetic valve use from 15%–40% in the 1990s to >70% of total prosthetic valve implantation in the 21st century (10, 11). Bioprosthetic valves have a propensity to structural valve deterioration and development of stenosis and/or regurgitation, especially in younger and active individuals. These valves were observed to fail due to the development of cusp calcification, tears, perforation, stretching, thickening, stiffening, and prolapse as the tissue degenerated over time (12). Thus, bioprosthetic valves were traditionally recommended for older patients with shorter life expectancy. However, with the advent of transcatheter technology and the use of valve-in-valve (ViV) therapy, bioprosthetic valves may offer interventional strategies that avoid redo operations.

On the other hand, mechanical valves present the well-known risk of thrombotic and hemorrhagic complications. The requirement of anticoagulation (AC) for all mechanical valves is a major barrier for many patients. Especially for older patients, it presents the increased risk of hemorrhage with falls, but for all patients, it requires the hassle of daily dosing and blood monitoring of Warfarin. Different studies have evaluated the possibility of alternate AC strategies. The PROACT study in 2013 showed that lower doses of Warfarin [test international normalized ratio (INR), goal 1.5–2.0] could safely be used 3 months post SAVR instead of standard Warfarin (INR goal 2.0–3.0) dosing (13). The PROACT Xa study went further to evaluate the possibility of using Apixaban for 3 months post SAVR; however, the study was terminated early as the Apixaban cohort was found to have higher rates of valve thrombosis and thromboembolic events (14). The RE-ALIGN study looked at the use of Dabigatran in mechanical valves (mitral and aortic), immediately and 3 months after SAVR, but it was also terminated early for higher risks of hemorrhage and thromboembolic events in the Dabigatran cohort (15).

Another major consideration for young patients requiring SAVR is the use of AC during pregnancy. There is a class 1 recommendation to undergo intervention prior to pregnancy in symptomatic women with severe valvular disease (8). However, uninterrupted AC with mechanical valves is essential throughout pregnancy due to the increased risk of thrombosis in this hypercoagulable state. Due to Warfarin's teratogenic effects, recommended strategies involve the use of low-molecular-weight heparin either throughout pregnancy or in the first trimester followed by Warfarin for the remaining two trimesters.

Several studies have compared outcomes of mechanical to bioprosthetic valves. In their review, Zhao et al. found no significant difference in survival between mechanical and bioprosthetic valves when looking at age groups 50–70 and 60–70; however, mechanical valves showed a survival benefit when patients less than 50 years old were included in the analysis (12). As expected, they also found higher AC and bleeding events but lower rates of reoperation in the mechanical valve group while the bioprosthetic valve group had higher structural valve degeneration (12). Similarly, Chang et al. found no difference in survival (30-day survival as well as actuarial 15 year survival) or stroke rates between the two groups; however, the bioprosthetic group had a higher cumulative incidence of reoperation over 15 years (12% compared to 6.9% in the mechanical valve group) but a lower bleeding risk (6.6% compared to 13% in the mechanical group) (11). The 30-day mortality rate of reoperation on bioprosthetic valves has been reported at 5%–6% in some studies (16–18) and as high as 9% in another (11).

##### 2.1.1.2 Aortic root enlargement for patient-prosthesis-mismatch

Patient-prosthesis mismatch (PPM) occurs when a prosthetic valve size is inappropriate for the patient's cardiac output and creates a high gradient across the left ventricular outflow tract. It is defined as a ratio of effective orifice area to body surface area (EOA/BSA) of less than 0.8 cm^2^/m^2^ (moderate = .65–.85, severe = less than .65). To avoid PPM, the patient's age, BSA, and level of activity must be considered in selecting a valve size. PPM increases the risk of early stenosis-structural valve degeneration, and has even been associated with increased all-cause and cardiac-related mortality following SAVR (19, 20).

Aortic root enlargement is a preemptive step that can be utilized in patients at increased risk of PPM to allow for a larger prosthesis valve size. Studies comparing isolated SAVR with or without root enlargement have found no difference in operative mortality or complication rates despite longer cross-clamp and bypass times; however, the former was associated with lower risk of significant PPM (21, 22). Similarly, Yousef et al. compared patients who underwent SAVR with root enlargement to those who underwent SAVR alone and found that those undergoing root enlargement were more likely to undergo the implantation of a smaller prosthesis size but the iEOA did not differ between the groups (23). Hence, these patients who were undergoing root enlargement were bound to receive an even smaller valve size with negative consequence on their iEOA. They also found root enlargement was utilized more in younger patients and in women. Root enlargement allows patients with smaller roots to undergo implantation of a larger sized prosthetic valve to safely decrease risk of PPM, but also to allow future ViV procedures.

#### 2.1.2 Aortic valve repair

The necessity of anticoagulation with mechanical valves and poor durability of tissue valves raises the argument for preserving the native aortic valve with repair. The type of repair used depends on the mechanism of valvular insufficiency. Just as Carpentier applied a system to assess mitral valve disease, he projected a similar functional framework on aortic regurgitation: type I describes normal leaflet motion with poor leaflet coaptation due to annular dilation, leaflet perforation or vegetations, type II is due to excessive leaflet motion, and type III with restricted leaflet motion (Table 1) (24). Carpentier recommended different repair techniques depending on etiology and studies have shown different outcomes depending on the type of repair. As in mitral repairs, the technical details and judgment on which approach to use for aortic repair veers more into the art rather than the science of surgery that can be hard to reproduce or study.

**Table 1:** Aortic valve repair techniques.

Type	Leaflet motion	Leaflet structure	Aortic STJ, ascending pathology	Common etiologies	Repair techniques
Type I	Normal	Often abnormal, leaflet fenestration, perforation	Often dilated	Annular dilation, aortic aneurysm, leaflet perforation or fenestration from endocarditis	Valve sparing root replacement/Ascending aortic replacement, patch repair of perforations
Type II	Excessive	Normal	Normal	Leaflet prolapse, flail leaflet due to excessive tissue, commissural disruption in aortic dissection	Leaflet edge plication, free margin resuspension
Type III	Restricted	Thickened, fused leaflets common	Normal	Calcific valvular disease, fibrosis of leaflets, bicuspid valve, rheumatic disease	Leaflet shaving, leaflet extension with pericardial patch, Root/ascending replacement

Type I insufficiency is commonly the consequence of active or healed endocarditis. Although primary repair of leaflet perforations, fenestrations, or tears remains the ideal approach, the extensive excision of vegetations often depletes native tissue, rendering reconstruction challenging. Some surgeons use bovine pericardial vs. autologous pericardial patch fixed with glutaraldehyde and a monofilament suture to repair any fenestrations or tears (25). Unfortunately, the data has consistently shown that patch augmentation, regardless of the material, is prone to failure (26).

For excessive leaflet motion or prolapse (type II), the leaflet edges can be plicated with a polypropylene suture (27). This is most effective when one cusp is prolapsing as the surgeon can use the coaptation height of the other functional leaflets as a reference point. If all three are dysfunctional, one can use the midpoint of the sinuses as the ideal coaptation height. El Khoury et al. also advocate for free margin resuspension in redundant leaflet tissue using two 7-0 PTFE sutures which are run from the commissure, across the free margin, and tightened to improve coaptation.

Repair techniques for restricted leaflet motion (type III) are limited by durability. Excessive leaflet shaving can lead to retraction as well as scarring, and while leaflets can be extended with a bovine pericardial patch, outcomes are suboptimal (28, 29). Grinda et al. report a 90% freedom from valvulopathy at 7 years following repair for rheumatic AI (29). Their census included 89 patients with a mean age of 16. They reported 2 perioperative mortalities, 27 (30%) patients with residual grade I-II AI immediately post-operatively which was increased at follow-up to 44%, as well as 2 early failures of repair and 7 failures at 5-year follow up. Pathology from the late failures demonstrated progression of rheumatic disease with worsening fibrosis and retraction of the tissues. The high rate of residual AI and failures are among the reasons repair is generally not recommended for rheumatic disease. For non-rheumatic disease, pericardial extension has better outcomes. However, type III AI in general has been associated with the lowest durability with a 5 year reoperation risk of 15% (30). The high surgeon technical variability as well as the short study period and single center data set does make it challenging to study the outcomes of repair techniques.

The literature better supports bicuspid aortic valve repair due to its greater reproducibility than tricuspid. Bicuspid valves can be divided into symmetric and asymmetric phenotypes in which the former has commissures which are closer in alignment with a typical tricuspid valve at 120° compared to 180°—repairs for the latter typically have better outcomes (30). Sievers classification for bicuspid aortic valve includes three types: type 0 lacks a median raphe (a cusp fusion) and comprise of two symmetric aortic sinuses and two commissures; type 1 includes a fusion of the left and right cusps with a pseudo-commissure; and lastly, type 2 describes a valve with two raphes (31). Type 0 valves are frequently associated with prolapse of the valve and benefit from plication or resuspension. The more common pathology is type 1 which also comprises 80% of repaired valves (30). The leaflet motion is often restrictive, so the raphe can be resected and then the remaining tissue is reapproximated with running locking suture to prevent purse-stringing or excessive thrombogenic knots on the valve surface (32, 33). The need for cusp repair, particularly due to calcification, is an independent risk factor for late failure; however, overall outcomes for bicuspid aortic valve repairs have shown a 10-year freedom from reintervention rate that is greater than 80% (34).

##### 2.1.2.1 Repair vs. replacement

The number of options available to patients with AI has dramatically increased in the modern era. The reduced risk of prosthetic infection and lack of anticoagulation requirement are weighed against the question of durability with repairs. One study examined the German aortic valve registry which included 2,327 patients who underwent aortic valve repair (29%) and replacement (71%) for AI (35). They demonstrated 97.7% survival in those who underwent repair as compared to 96.4% who underwent replacement at 1 year. They also found no difference in rates of reintervention for the aortic valve. Repair had the benefit of reduced residual gradients with 3% of patients demonstrating a gradient >20 mmHg. Repair was also associated with greater quality of life, mobility and reduced duration of unemployment. The study was biased towards success for repair as it excluded cases which were converted from repair to replacement. These comparisons must be viewed with the understanding that the pathology of valves that can be repaired and those that must be replaced are inherently different.

All prosthetic valves—mechanical, tissue, or transcatheter aortic valve replacements (TAVRs)—carry a risk of prosthetic infection which ranges from 0.3%–1.2% per patient-year for surgical valves vs. 0.6%–3.4% for TAVR (36). Repair offers a lower risk of prosthetic valve endocarditis which typically calls for immediate surgical removal and replacement of the valve. Additionally, mechanical valves call for lifelong anticoagulation with coumadin which can be burdensome with regular testing and carries a high risk for bleeding complications which are less tolerated in older populations. Repair techniques however are highly variable, depend on the etiology of the valve insufficiency, and are subject to the operator's technical skills. Given the advantages of valve repair, the option should be offered when reasonable.

#### 2.1.3 Ozaki (aortic valve neocuspidization) technique

The Ozaki procedure involves the replacement of the diseased aortic valve leaflets with autologous or bovine pericardium. Among the advantages is the ability to maximize the valve orifice area—since there is no stented frame involved—and to allow for a large coaptation area (37). Though introduced in adults, it has a higher rate of use in pediatric patients nowadays, especially in patients who cannot undergo the Ross procedure because of truncus arteriosus or AI with severely dilated aortic annulus (38). Further investigation is required to evaluate its long-term outcomes, but small studies so far have demonstrated reasonable findings. Ozaki et al. reported freedom from reoperation of 96.2% at 53 months of follow-up in 404 patients with bicuspid, unicuspid, or quadricuspid valves of mean age of 69 years (39). Wiggins et al. reported freedom from reoperation or moderate and greater AI of 79% at 3 years in 58 patients with a median age of 14.8 years (40). While Baird et al. reported freedom from moderate or greater AI and moderate or greater AS of 88% at 2 years in 57 patients with median age of 12.4 years (41).

#### 2.1.4 Ross procedure

**Figure 1:**
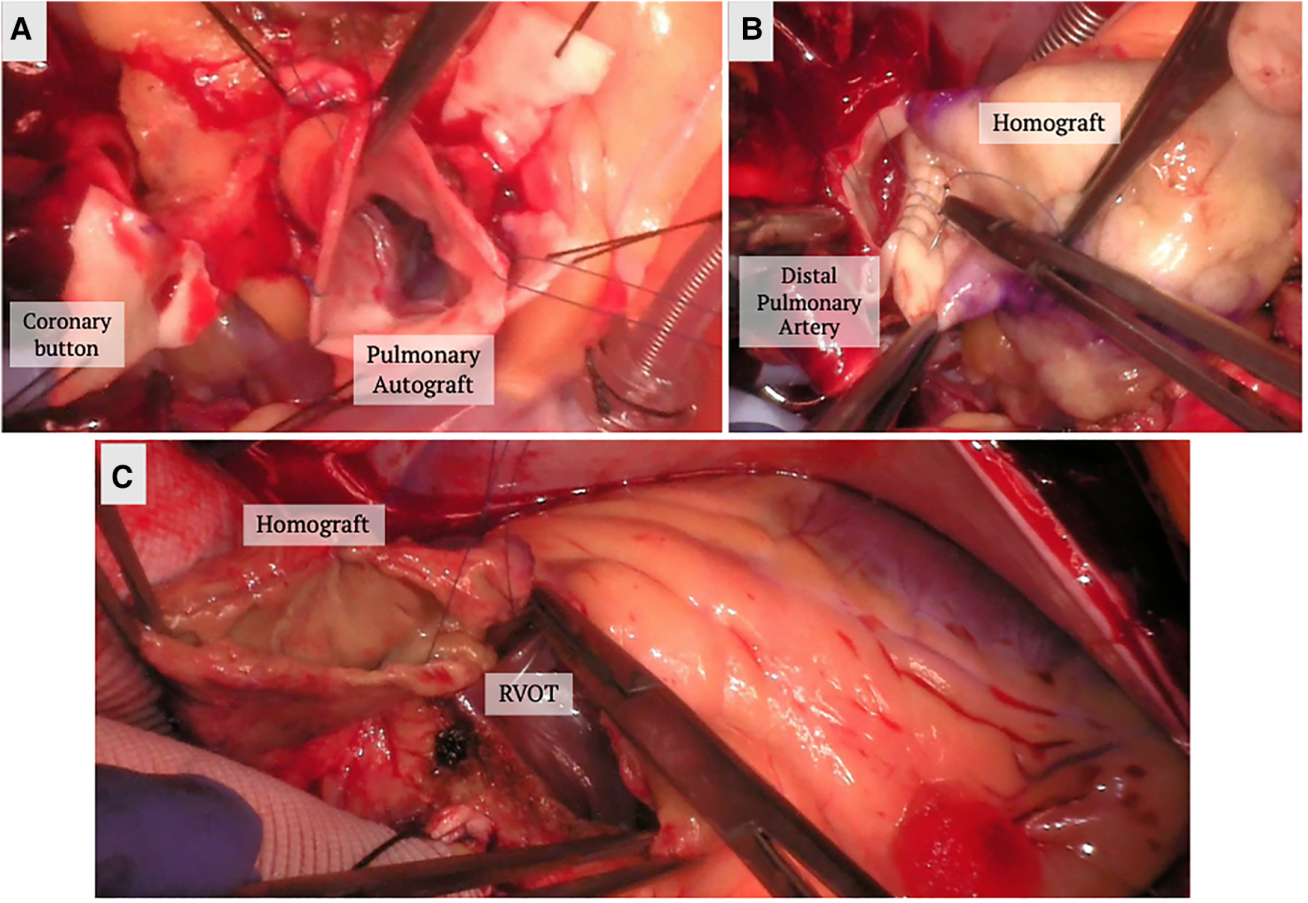
Ross procedure. (**A**) The pulmonary autograft is dissected and reimplanted in the left ventricular outflow tract. The coronary buttons are prepared and reimplanted in the autograft. (**B**) A homograft is implanted in the position of the pulmonary valve position. (**C**) The homograft is secured to the right ventricular outflow tract.

The Ross procedure involves the replacement of the diseased aortic valve with the patient's native pulmonic valve, followed by the replacement of the native pulmonic valve by a pulmonic homograft (Figure 1). The technique is thought to provide improved hemodynamics, and protection against infection and thrombosis due to the living nature of the prostheses (38). When comparing conventional SAVR with the Ross procedure, studies have found no difference in perioperative or 30-day mortality (42, 43), some studies even found decreased late mortality at a mean follow up of 2.6 years (43). When looking specifically at long-term survival, El-Hamamsy et al. found survival rates of 98%, 97%, 95% and 95% at years 1, 5, 10, and 13 post Ross, respectively, vs. 96%, 92%, 83% and 78% in patients who had undergone aortic homograft replacement (44). They also found the survival rate in the Ross group was similar to that of the age and sex-matched general population (99%, 98%, 97% and 95% at years 1, 5, 10, and 13) (44). Other studies have also shown similarly promising long-term survival rates (95.6%, 91.8%, 86.3% and 80.5% at five, ten, fifteen and twenty years, respectively) (45).

In addition, studies have found lower complication rates of stroke and bleeding in patients who underwent the Ross in comparison to conventional SAVR, as well as improved quality of life when compared to mechanical SAVR (42, 43). The crux of the Ross procedure is in the reoperation rate, especially at the pulmonic homograft, with some studies showing minimal difference while others noting a 50% rate of echocardiographic evidence of dysfunction at 20 years post implantation (42–46). Of note, current recommendations are for the performance of the Ross procedure at specialized centers, as nationally based series have shown increased complication and mortality rates when compared to conventional SAVR at non-specialized centers (47).

Other safeguards have been placed on the use of the Ross procedure for AI because of associated worse outcomes. For example, familial aortopathy and connective tissue disorder are contraindications for the procedure due to increased risk of long-term failure and dilatation. Similarly, preoperative aortic insufficiency has been found to be an independent predictor of reoperation (46). Ryan et al. compared the outcomes of the Ross procedure in patients with aortic stenosis (AS) vs. AI at a single center (48). While they found no difference in the mortality rate, autograft dilation was seen in 15% of patients with AI vs. 1.5% in AS and the odds ratio for autograft reoperation on patient's AI vs. AS was 10.7 times. Therefore, the use of the Ross procedure in patients with pure AI is controversial and generally not recommended.

### 2.2 With presence of aortic root dilation

#### 2.2.1 Composite valve aortic root replacement (modified bentall procedure)

**Figure 2:**
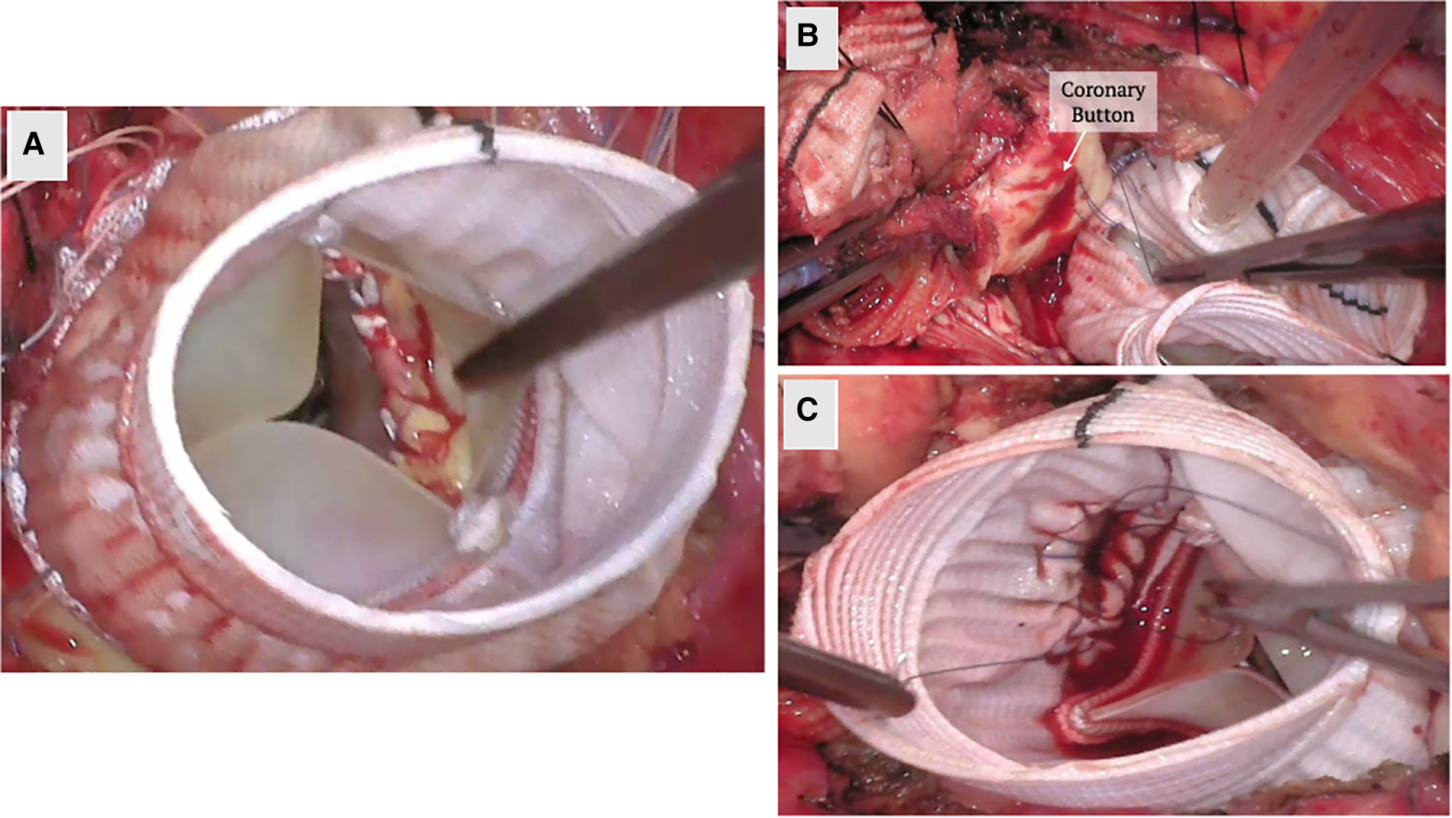
Biobentall procedure. (**A**) An aortic tissue valve and root replacement with a composite graft (Bio-Bentall) is demonstrated here. Once the aorta is opened and the aortic valve is excised, the composite graft is implanted into the annulus. (**B**) The coronary buttons are created from the sinuses of Valsalva and remaining tissue is excised. (**C**) The coronary buttons are sewn into the graft as demonstrated.

For patients with AI who require concurrent aortic valve and root replacement, the modified Bentall procedure allows for composite valve and graft replacement in lieu of repair. In this technique, the valve is excised, sinuses resected, coronary buttons prepared for side to end anastomosis, and the conduit inserted at aortic annulus up to where aortic disease ends. It traditional involves the use of a mechanical valve; however, bioprosthetic valves can also be used in the “Biobentall” technique (Figure 2). This technique has become widespread, especially in the era of commercially available conduit grafts (e.g., Onyx valve composite and KONECT RESILIA aortic valved conduit).

Mechanical composite graft root replacement has shown reassuring outcomes, even showing a 98% freedom from re-intervention at the root in 15 years (49). In comparing early outcomes of mechanical and biological valve composite root replacement, the composite mechanical root cohort was found to have a higher perioperative rate of bleeding; however, there was no statistically significant difference in mortality, endocarditis, re-operation or stroke rates (50). However, studies looking at long-term outcomes for Biobentall have noted a higher re-operation rate (51). When comparing outcomes of compositive grafts with VSARR, the latter has shown lower rates of late mortality and complications without a statistically significant difference in early outcomes (51–53).

#### 2.2.2 Valve-sparing aortic root repair (VSARR)

The aortic valve functionally relies on the root apparatus, specifically the sinuses of Valsalva, in which blood coils against the sinus walls creating eddy currents that promote leaflet closure and coaptation during end-systolic deceleration of blood flow (54, 55). Effacement of the sinuses and dilation of the root can interfere with this physiology contributing to aortic insufficiency. Dilation of the sinotubular junction (STJ) can also pull on the commissures of the aortic valve leaflets creating a distance between their free margin, leading to poor coaptation and central regurgitation (56). Though, at times, there is an isolated dilated ascending aorta without involvement of the root—which can also contribute to poor aortic valve function—, frequently there is involvement of the root. While initially the standard approach was to replace the valve and root along with other aneurysmal portions of the thoracic aorta, increased recognition of the physiology above has led to the development of two forms of valve-sparing root aneurysm replacement techniques.

Magdi Yacoub was the first to propose reconstructing the aortic root with the restored functional anatomy to resolve AI (57, 58). He proposed isolating the commissures and reconstructing neo-sinuses of valsalva sewn around the commissures. The coronary buttons are then reimplanted into the neosinuses. Correction of the STJ dilation or often the asymmetric sinus dilation can lead to improved coaptation (59).

**Figure 3:**
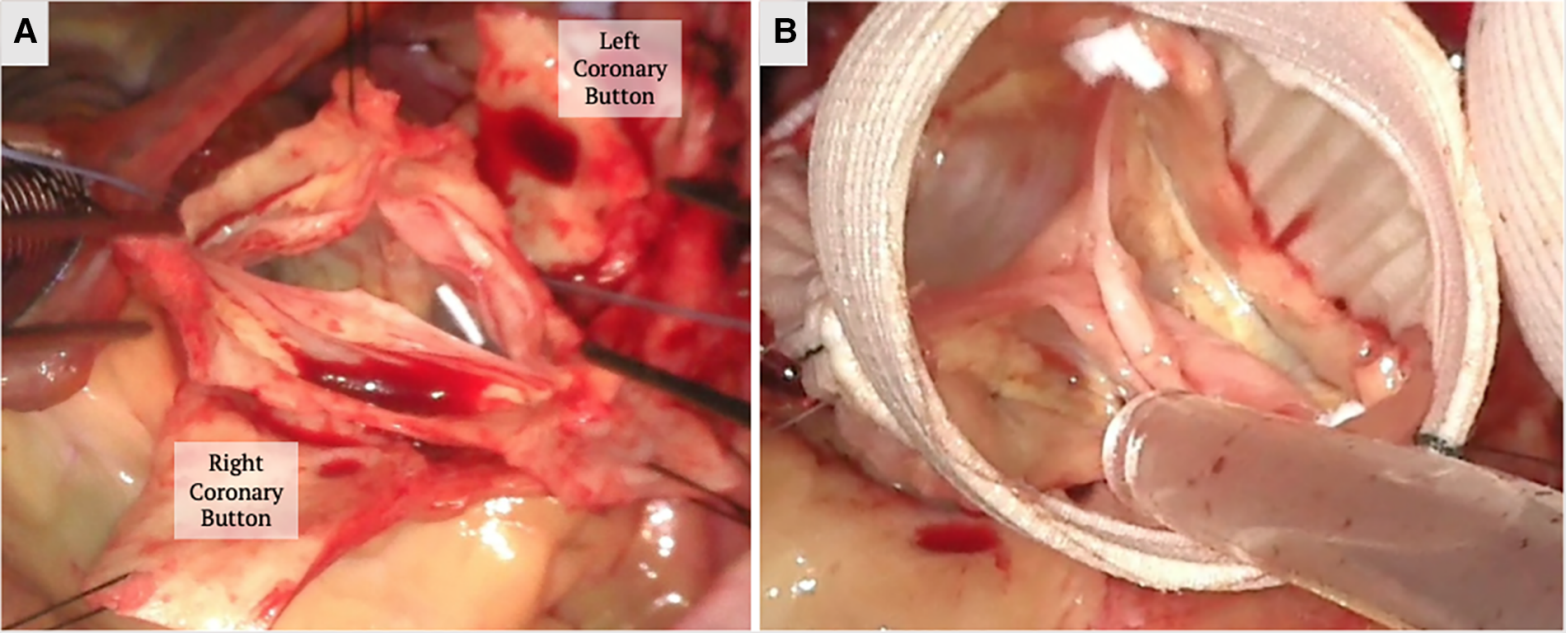
A valve sparing aortic root replacement (David procedure) is shown above. (**A**) The aortic root is dissected down to the annulus and the commissures are isolated with stay sutures to mark their orientation. The coronary buttons are created. (**B**) The annulus, commissure heights and free margin of the leaflets are measured to select the graft size and the commissures are reimplanted within the Valsalva graft. The height of the commissures are adjusted for optimal leaflet coaptation. Coronary buttons are reimplanted and the valve is tested for competency. The images above demonstrate a trileaflet aortic valve.

Shortly after, Tirone David and Chris Feindel published their reimplantation technique (Figure 3) (60). The benefit of their approach is the anchoring of the new graft into the annulus and resuspension of the commissures within the graft. This secures and reduces the annular dimensions, which becomes critical in patients with annular dilation or connective tissue disease. Once the root is dissected and the commissures isolated, a graft is chosen based on the STJ diameter, height of the cusps, and the commissures. The height of the commissure is used as an approximation of the ideal STJ diameter with the addition of 4 mm to account for aortic wall thickness. Next, the commissures are sutured along the wall of the graft reconstructing the STJ, and the coronary arteries are reimplanted on the neosinuses (Figure 3). There are new grafts that have entered the market that resemble the natural outpouchings of the sinuses of Valsalva; there are also new modifications to this procedure used to recreate the outpouchings in the artificial grafts (61, 62). The Stanford modification entails attaching a smaller graft to a larger graft to mimic the sinuses (62). David also tailored his technique by plicating the STJ to mimic the natural anatomy.

The weakness of Yacoub's remodeling technique is largely due to ineffective correction of the ectatic annulus. Preoperative annular dilation exceeding 28 mm significantly increases reintervention risk, reaching 37% in one study, due to greater risk of recurrent AI (63–65). Lansac attempted to compensate for this flaw by supplementing the procedure with an annuloplasty using an expandible aortic ring (66, 67). This was studied in a group of 747 patients who underwent remodeling repair with 295 patients who had the supplemented annuloplasty. Though this helped reduce five year recurrence of severe AI (freedom from AI at 5 years from 81%–92% in bicuspid aortic valves (*P* = .8), and 93%–98% in tricuspid aortic valves (*P* = .6), the difference was not statistically significant and the procedure did not affect 5 year freedom from reoperation (65). Conversely, the group at Duke has proposed using an internal annuloplasty ring “HAART” with the remodeling technique in bicuspid and tricuspid valves. They use a rigid ring that is implanted along the commissures in the same manner as a prosthetic aortic valve. The reasoning is that this provides the support of a suture annuloplasty but does not dilate like previous techniques (68, 69). Short term follow up results are promising (5 months for bicuspid valves and 11 months for tricuspid) with no reoperations or aortic insufficiency. Interestingly, the early post repair mean gradient was 18 mmHg and 7 mmHg for tricuspid and bicuspid valves, respectively. However, the presence of the ring may make transcatheter valve-in-ring options prohibitive in the future if the valves develop further deterioration.

Due to the above data, the reimplantation technique is preferred for patients with a dilated aortic root, genetic aortopathy with a predilection towards aneurysm formation, and bicuspid aortic valves (70, 71). Three studies have evaluated the long-term outcomes of the reimplantation techniques, showing greater than 94% freedom from reoperation and recurrent aortic regurgitation at 5 years, and greater than 87% at 10 years (72–74). Valve-sparing aortic root replacement for type A dissections with associated aortic insufficiency is a controversial procedure due to the life threatening nature of the condition. Opponents argue that the procedure is longer, more complex and can contribute to worse perioperative mortality. Most data on long-term outcomes of the reimplantation technique include type A dissections and no significant differences in mortality or bleeding rates. However, the remodeling technique was shown to have a higher failure rate for acute type A dissections (75). It may be that patients prone to developing aortic dissections or already affected by cystic medial necrosis have intrinsic tissue quality that is of poor quality and leaving unsecured commissural tissue can predispose to subsequent degeneration.

To simplify the procedure and reduce operative time, the Florida sleeve technique has been developed as an option for the remodeling technique in which the coronary arteries are left intact. The technique involves the placement of a graft with keyhole incisions to accommodate the coronary ostia over the sinuses down to the aortic root. It is preferred for its simplicity especially in long cases such as type A dissections. Initial studies have shown acceptable outcomes (76, 77).

#### 2.2.3 Isolated root remodeling

With isolated annular dilation, some have attempted various annuloplasty techniques to help leaflet coaptation for functional AI along the model of mitral and tricuspid repair techniques. In fact, aortic annuloplasty predates prosthetic valve replacement (78). Lack of widespread adoption is due to the early failures of these techniques (79). Cabrol proposed an internal suture annuloplasty with 2-0 prolene, and though it was initially used by many, it had poor long-term outcomes with high recurrence rates. The Lansac external ring has been used in this setting, however, due to difficult dissection into the ventriculoaortic junction, placement in the right plane can be prohibitively challenging.

## 3 Transcatheter management of aortic insufficiency

TAVRs are becoming valid options for patients with aortic valve stenosis or mixed disease (AS and AI) regardless of their risk profile. Calcifications of the aortic valve leaflets represent a solid tissue substrate where the transcatheter heart valves (THV) can land and anchor. Development of newer generation valves with wrap-around skirts and more efficient profiles, allows for optimization of results with high technical and device success rates, and low incidence of paravalvular leak (PVL) and other device related complications. If recent data from randomized control trial (RCT) shows non-inferiority of TAVR compared to surgery in the mid-term in patients with AS, the same statement cannot be said about transcatheter treatment of AI. The 2020 ACC/AHA Guidelines for management of valvular heart disease do not recognize TAVR as an option in patients with AI, and in patients that have indication for surgery, TAVI is considered harmful and should not be performed (COR 3, LOE B) (8). Nevertheless, there is an increasing interest of transcatheter options in patients with AI, especially if they are not candidates for any surgical procedure. Data available so far originates from small series, with adoption of available THV not designed for regurgitant valves, and adapted to variate anatomies as palliative last resort solutions. Anatomical characteristics of the aortic root and design and mechanism of deployment of TAVR valves represent significant limitations in patients with AI. The lack of calcium, the frequently dilated aortic root, and the different root dynamics associated with AI, including the “suction effect” from regurgitation, make the placement of commercially available THVs challenging. Rates of valve migration, PVL, new PPM, and need for second interventions remain high, and represent some of the limits that operators face in patients with pure AI.

One of the main concerns of the use of THVs in AI is anchoring. Different valves designs offer sealing at different levels of the aortic root/ascending aorta. Self-expandable valves such as Evolut (Medtronic, Minneapolis MN, USA) and Acurate (Boston Scientific, Marlborough, MA, USA) anchor at both the annulus and the proximal ascending aorta, while others like balloon- and mechanically- expandable Sapien (Edwards, Irvine CA, USA) and Lotus (Boston Scientific, Marlborough, MA, USA) (the latter no longer available at the present time) anchor at the annular level only. The JenaValve (Irvine, CA, USA) is unique in its mechanism of anchoring, relying on stabilization at the level of both the leaflets and annulus.

The CoreValve's (Medtronic, Minneapolis MN, USA) fixation depends solely on radial expansion and oversizing. The early experience of the use of this THV in patients with AI showed the need for a second valve implantation in almost 1 out of 5 cases, >20% of patients left with more than mild AI, and a high PPM rate (80). The initial experience with the Acurate Neo TF system described the outcomes of 20 patients with pure AI among centers in Europe and Israel. Initial results were promising, device success was 90% and more than half patients had no AI at discharge; there was no reported mortality or stroke and a 15% PPM rate (81).

Yoon et al. collected data from 331 patients with AI, comparing outcomes of newer and older generation devices. Cardiovascular mortality at 1-year was 15.6%, higher in patients with post procedural residual AI (82). The newer THVs were superior to the old ones in terms of success rate and need for second valve implantation.

Poletti and colleagues recently published the results of an International retrospective study on placement of commercially available off-label THVs in patients with pure AI (83). Both self- and balloon- expandable valves were implanted in 201 inoperable patients with AI, and technical and device success, all-cause mortality and heart failure rehospitalizations were assessed at 1 year. The authors showed a technical- and device- success rates of 83.6% and 76% respectively, as well as high incidences of valve migration (12.4%) and residual greater than moderate PVL. Balloon expandable and self-expandable valves seemed to perform equally in terms of primary and secondary endpoints. One-year results showed a cumulative incidence of composite endpoints of 17.1% and a higher occurrence of all-cause mortality in patients who experienced valve migration. These results are clearly worse than the ones on THV implants in patients with AS, but they represent valid alternatives to medical therapy in patients who are not candidates for SAVR.

Of note, 10% of the patients included in the Pantheon Study were implanted with the JenaValve. The Trilogy platform is a supra-annular porcine pericardial THV specifically designed for treatment of regurgitant aortic valves. It is available in 3 different sizes (23–25–27 mm), and it is implanted via transfemoral access through an 18Fr. long sheath that reaches the level of the STJ. Peculiar features of this valve are the presence of 3 radial locators that allow anchoring to non-calcified leaflets and grant commissural alignment, the expanding nitinol frame resulting in low rates of PVL, and the large cells frame that ensures future coronary access. The JenaValve was implanted successfully in all patients, with no valve migration/embolization, no need for surgical intervention and/or second valve implantation.

The outcomes of the Pantheon Study are similar to the outcomes described by Adam and Colleagues in the first European experience of the Conformité Européenne approved Trilogy system in Germany (84). Fifty-eight consecutive patients with AI were included in the registry and implanted with the JenaValve THV. Device success was achieved in almost all patients, no moderate or moderate-severe residual AI was seen, and all valves showed excellent hemodynamics. The authors reported no case of valve migration, and none of the patients underwent salvage surgery for device -related complications. Thirty-day mortality remained low (1.7%), no stroke was observed, and the majority of patients improved their functional status. As previously described in other series, the need for PPM remained a major concern (19.6%).

The ALIGN-AR was a prospective, multicenter, single-arm study and looked at the safety and effectiveness of the JenaValve in high-risk patients with symptomatic moderate-severe and severe AI (85). Three-hundred and seventy-nine patients were screened and 177 were successfully implanted. Most of the cases were performed under general anesthesia. Intraprocedural mortality was 0% and technical and device success was achieved in 95 and 96.7% of the cases. There were only 4 total valve embolizations. All-cause mortality at 30 days was 2.2%, rate of disabling strokes was 1.1%, and total of primary safety endpoints of 26.7%, mainly driven by a PPM rate of 24%. Interestingly, new insights and changes in the implantation technique, reduced the PPM need to only 14% in the last third of treated patients. Hemodynamics were described to be excellent throughout a year, and incidence of PVL greater than mild was uncommon. Overall, the results of the ALIGN-AR trial were promising, as the Trilogy THV met the 30-days primary safety outcomes as well as the one-year primary efficacy outcome for all-cause mortality.

The J-Valve system (JieCheng Medical Technology Co, Ltd, Suzhou, China) is another device under investigation for the treatment of AI. It is a porcine valve on a self-expanding support frame and an overlying 3-prong clasper that is intended for a two-step deployement (86, 87). The claspers are placed in the aortic sinuses in the first stage to align positining as well as anchor the native valve leaflets to the support frame. This is followed by adjustment of the prothetic valve for ideal positioning before final deployement. A 2021 study comparing outcomes of its use in patients with AS vs. AI found comparable clinical and echocardiographic outcomes (88).

In conclusion, AI carries a multitude of uncertainty. There is a large proportion of patients with pure AI that are not ideal surgical candidates and lack of valid treatment options; conventional THVs approved for treatment of AS can be utilized in selected cases of AI, knowing that short- and mid-term results are inferior than the ones achieved in patients with AS. Third: the development of dedicated new devices and technologies could be the answer to the unmet need for less invasive non-surgical treatment of AI.

## 4 Conclusion

Current technology has allowed for a widespread avenue for the management of aortic valve insufficiency. These include surgical valve replacement, surgical repair, and transcatheter interventions. The unique qualities of AI have historically limited certain options, such as the Ross procedure and transcatheter options. Replacements with mechanical or bioprosthetic valves remain well established strategies, and still represent the gold standard of treatment of patients with AI. Refined surgical techniques, broader experience of specialized aortic surgeons and the advent and development of transcatheter devices, are re-designing the future of valve therapy, offering better long-term outcomes and avoidance of life-long anticoagulation. Preservation of the patients' native leaflets and repair of aortic roots should be the preferred option, especially in younger and healthier patients, but such complex operations should be deferred in specialized centers and in the hands of experienced aortic surgeons. The introduction of dedicated THV for the treatment of AI is promising, but the establishment of these devices in the daily practice will require further investigations.
